# Hypoxia Induced by Cobalt Chloride Triggers Autophagic Apoptosis of Human and Mouse Drug-Resistant Glioblastoma Cells through Targeting the PI3K-AKT-mTOR Signaling Pathway

**DOI:** 10.1155/2021/5558618

**Published:** 2021-05-27

**Authors:** Yuan-Wen Lee, Yih-Giun Cherng, Shun-Tai Yang, Shing-Hwa Liu, Ta-Liang Chen, Ruei-Ming Chen

**Affiliations:** ^1^Anesthesiology and Health Policy Research Center; Department of Anesthesiology, Taipei Medical University Hospital, Taipei Medical University, Taipei 110, Taiwan; ^2^Department of Anesthesiology, School of Medicine, College of Medicine, Taipei Medical University, Taipei 110, Taiwan; ^3^Department of Anesthesiology, Shuang Ho Hospital, Taipei Medical University, New Taipei City 235, Taiwan; ^4^Department of Neurosurgery, Shuang Ho Hospital, Taipei Medical University, New Taipei City 235, Taiwan; ^5^Graduate Institute of Medical Sciences, College of Medicine, Taipei Medical University, Taipei 110, Taiwan; ^6^Institute of Toxicology, College of Medicine, National Taiwan University, Taipei 100, Taiwan; ^7^Cell Physiology and Molecular Image Research Center; Department of Anesthesiology, Wan Fang Hospital, Taipei Medical University, Taipei 116, Taiwan; ^8^TMU Research Center of Cancer Translational Medicine, Taipei 110, Taiwan

## Abstract

Glioblastoma multiforme (GBM) is the most aggressive brain tumor. Drug resistance mainly drives GBM patients to poor prognoses because drug-resistant glioblastoma cells highly defend against apoptotic insults. This study was designed to evaluate the effects of cobalt chloride (CoCl_2_) on hypoxic stress, autophagy, and resulting apoptosis of human and mouse drug-resistant glioblastoma cells. Treatment of drug-resistant glioblastoma cells with CoCl_2_ increased levels of hypoxia-inducible factor- (HIF-) 1*α* and triggered hypoxic stress. In parallel, the CoCl_2_-induced hypoxia decreased mitochondrial ATP synthesis, cell proliferation, and survival in chemoresistant glioblastoma cells. Interestingly, CoCl_2_ elevated the ratio of light chain (LC)3-II over LC3-I in TMZ-resistant glioblastoma cells and subsequently induced cell autophagy. Analyses by loss- and gain-of-function strategies further confirmed the effects of the CoCl_2_-induced hypoxia on autophagy of drug-resistant glioblastoma cells. Furthermore, knocking down HIF-1*α* concurrently lessened CoCl_2_-induced cell autophagy. As to the mechanisms, the CoCl_2_-induced hypoxia decreased levels of phosphoinositide 3-kinase (PI3K) and successive phosphorylations of AKT and mammalian target of rapamycin (mTOR) in TMZ-resistant glioblastoma cells. Interestingly, long-term exposure of human chemoresistant glioblastoma cells to CoCl_2_ sequentially triggered activation of caspases-3 and -6, DNA fragmentation, and cell apoptosis. However, pretreatment with 3-methyladenine, an inhibitor of autophagy, significantly attenuated the CoCl_2_-induced autophagy and subsequent apoptotic insults. Taken together, this study showed that long-term treatment with CoCl_2_ can induce hypoxia and subsequent autophagic apoptosis of drug-resistant glioblastoma cells via targeting the PI3K-AKT-mTOR pathway. Thus, combined with traditional prescriptions, CoCl_2_-induced autophagic apoptosis can be clinically applied as a *de novo* strategy for therapy of drug-resistant GBM patients.

## 1. Introduction

Glioblastoma multiforme (GBM) is the most malignant brain tumor. In the clinic, GBM patients are regularly cured with standard surgical resection and successive concurrent chemoradiotherapy [1]. Temozolomide (TMZ) is the first-line chemotherapeutic drug for GBM [2]. Unfortunately, more than 50% of GBM patients will ultimately exhibit drug resistance and recurrence [3]. Because GBM develops in the brain, this cerebral location limits neurosurgeons' performance of completely removing tumors [4]. Moreover, glioblastoma cells possess unique features of rapid proliferation, migration, and invasion [5]. Following surgery, residual glioblastoma cells existing on the periphery of a brain tumor can speedily proliferate and invade other areas to recur as more-aggressive brain tumors [4]. As a result, GBM patients usually have very poor prognoses. Even if patients are energetically cured, their average survival is only 12~18 months [6]. Until now, chemoresistance is still a key challenge for therapy of glioblastomas. Therefore, establishing *de novo* strategies to overwhelm drug tolerance by GBM is an emergent and necessary issue.

Mammalian cells require an adequate supply of oxygen for energy production in order to support cell activities and functions. Hypoxia is a condition in which there is an insufficient oxygen source in a region of the body [7]. Being related to physiological and pathological situations, hypoxia is highly associated with human health and diseases, especially in the brain [8, 9]. Throughout its entire lifespan, the human brain is often threatened by cerebral hypoxia [10]. For example, prenatal hypoxia that occurs in a key stage of brain formation may cause morphological variations in brain structures that are involved in learning and memory and ultimately affect development of cognitive functions. In ischemic brain diseases, hypoxia can directly disrupt the integrity of the blood-brain barrier (BBB), thus leading to vasogenic edema, brain swelling, and neuronal injury [11]. Moreover, cerebral hypoxia can also be induced by certain diseases, such as asthma, that interfere with breathing and blood oxygenation [12]. More attractively, hypoxia is also detected in solid cancers, particularly in brain tumors [13]. Hypoxic conditions may induce insults to glioblastoma cells. Otherwise, in response to hypoxic stress, glioblastoma cells can produce and excrete vascular endothelial growth factor (VEGF) to stimulate neovascularization from preexisting blood vessels [14]. In response to hypoxic stimuli, hypoxia-induced factor- (HIF-) 1*α*, a subunit of heterodimeric HIF-1, can be significantly upregulated and then functions as a representative transcription factor to regulate downstream gene expressions [9, 15]. A number of studies disclosed the complexity and importance of the HIF-1*α* signaling pathway in hypoxia [16]. Accordingly, HIF-1*α* and its downstream targets are emerging as novel therapeutic options for treating brain tumors.

Autophagy, a process of self-degradation and catabolism, is generally considered to be a survival mechanism in response to nutrient insufficiency-induced stress [17]. In addition, autophagy participates in preventing certain diseases, such as cancer, neuronal disorders, cardiomyopathy, diabetes, liver disease, autoimmune diseases, and infections, by engulfing damaged organelles and intracellular ribosomes and protein aggregates into double-membraned autophagosomes. Hypoxia can induce cell autophagy [18]. In the hypoxic tissue microenvironment, adenosine monophosphate- (AMP-) activated protein kinase (AMPK) is activated due to an increase in the ratio of intracellular AMP and adenosine triphosphate (ATP) [19]. Subsequently, activated AMPK can trigger cell autophagy through directly inducing autophagy-associated light chain 3 (LC3) and indirectly suppressing activity of the mammalian target of rapamycin (mTOR) [13, 20]. When oxygen deprivation occurs in the tissue microenvironment, HIF-1*α* is proximately induced and then activated in response to hypoxic stress [21]. HIF-1*α* can induce cell autophagy via inducing BNIP3 and LC3 expressions [22]. Traditionally, hypoxia-induced autophagy is thought to promote tumor resistance [13]. In addition to autophagy, hypoxic conditions can induce cell apoptosis and necrosis in follicles of mammalian ovaries [18]. Our previous study showed that honokiol, an anticancer drug, induces autophagic insults to neuroblastoma cells via activation of the phosphoinositide 3-kinase- (PI3K-) AKT-mTOR and endoplasmic reticular (ER) stress/extracellular signal-regulated kinase (ERK)1/2 signaling pathways [23]. Moreover, a longer period of treatment with honokiol led to autophagy and the death of glioblastoma cells [24]. Our previous study also demonstrated that cobalt chloride (CoCl_2_), an inducer of HIF-1*α*, can be used as a chemical hypoxia model to induce autophagic death of human glioblastoma cells via a p53-dependent mechanism [25]. More than 50% of GBM patients ultimately exhibit chemoresistance, and drug-resistant glioblastoma cells highly defend against apoptotic insults [1]. In this study, we successfully isolated human and mouse TMZ-resistant glioblastoma cells as our experimental models to investigate whether or not a prolonged administration of hypoxia could induce autophagic killing of drug-resistant glioblastoma cells and the possible action mechanisms, focusing on the PI3K-AKT-mTOR signaling pathway.

## 2. Materials and Methods

### 2.1. Selection and Culturing of Human and Mouse Drug-Resistant Glioblastoma Cells

TMZ-sensitive human U87 MG and mouse GL261 cells were used for selection of drug-resistant U87 MG-R and GL261-R glioblastoma cells as described previously [26]. In brief, U87 MG and GL261 cells were seeded in 12-well tissue culture plates at a density of 10^5^ cells per well and maintained in Dulbecco's modified Eagle's medium (DMEM; Gibco-BRL Life Technologies, Grand Island, NY, USA) with 10% fetal bovine serum, 100 *μ*g/ml streptomycin sulfate, and 100 U/ml penicillin and cultured in a humidified incubator with 5% CO_2_ at 37°C. Glioblastoma cells were treated with 50 *μ*M TMZ for 2 days. Later, human U87 MG and mouse GL261 cells were trypsinized and diluted 0.2~1.0-fold. Diluted cells were cultured in DMEM with 100 *μ*M TMZ. Surviving cell colonies were dissociated with trypsin and further grown in culture medium containing 100 *μ*M TMZ. After sequential selection of drug-resistant glioblastoma cell colonies, TMZ-tolerant U87 MG-R and GL261-R cells were successfully selected. Human normal astrocytes (HA-h) purchased from ScienCell Research Laboratories (Carlsbad, CA, USA) were cultured in astrocyte medium (ScienCell Research Laboratories).

### 2.2. Creation of Hypoxic Conditions and Drug Treatment

Hypoxic conditions in drug-resistant U87 MG-R and GL261-R glioblastoma cells were created by inducing HIF-1*α* expression following treatment with CoCl_2_ as described previously [25]. CoCl_2_, bought from Sigma (St. Louis, MO, USA), was freshly dissolved in phosphate-buffered saline (PBS), containing NaCl (0.14 M), KCl (2.6 mM), Na_2_HPO_4_ (8 mM), and KH_2_PO_4_ (1.5 mM). TMZ-resistant U87 MG-R and GL261-R glioblastoma cells were exposed to 100 *μ*M CoCl_2_ for 6, 12, and 24 h. Levels of HIF-1*α* were measured in order to confirm hypoxic conditions in drug-resistant glioblastoma cells. Control cells received PBS only.

### 2.3. Analyses of Cell Morphology and Survival

Morphologies and survival of human and mouse drug-sensitive and -resistant glioblastoma cells were analyzed according to a previously described method [27]. Drug-resistant glioblastoma cells (10^4^ cells/well) were seeded in 12-well tissue culture plates overnight. After drug treatment, cell morphologies were observed and photographed using an inverted light microscope (Nikon, Tokyo, Japan). Then, the cells were trypsinized with 0.1% trypsin-EDTA. After centrifugation, glioblastoma cells were suspended in PBS buffer and stained with a trypan blue dye. Fractions of living cells with white signals were visualized and counted with a light microscope (Nikon).

### 2.4. Examination of Cell Proliferation

Proliferation of human drug-resistant glioblastoma cells was assayed by analyzing the incorporation of bromodeoxyuridine (BrdU) into genomic DNA as described previously [28]. Glioblastoma cells at 3 × 10^3^ cells/well were seeded in a 96-well cell culture plate. Following CoCl_2_ treatment, replicating glioblastoma cells were reacted with 10 mM BrdU for a further 2 h. Then, human drug-tolerant glioblastoma cells were fixed with 4% paraformaldehyde. A cell proliferation enzyme-linked immunosorbent assay (ELISA) kit purchased from Roche (Mannheim, Germany) was used in this study to measure amounts of BrdU incorporated into genomic DNA of glioblastoma cells. Signals were read using a microplate photometer (Thermo Fisher Scientific, Tewksbury, MA, USA) and statistically analyzed.

### 2.5. Assay of Mitochondrial NAD(P)H Oxidoreductase Activity

A colorimetric method was carried out to examine activities of mitochondrial NAD(P)H-dependent oxidoreductase enzymes in human drug-resistant glioblastoma cells as described previously [29]. Briefly, human drug-resistant glioblastoma cells were seeded in 96-well cell culture plates at a density of 10^4^ cells/well for 12 h. After exposure to CoCl_2_, TMZ-tolerant glioblastoma cells were cultured with fresh DMEM containing 0.5 mg/ml 3-(4,5-dimethylthiazol-2-yl)-2,5-diphenyltetrazolium bromide for a further 3 h. The formazan products, metabolized by mitochondrial NAD(P)H oxidoreductases, were then dissolved in DMSO. Dark-brown signals were spectrophotometrically measured at 550 nm using a spectrophotometer (BioTek, Winooski, VT, USA).

### 2.6. Levels of Cellular ATP

A bioluminescence assay was conducted to measure levels of cellular ATP in human TMZ-resistant glioblastoma cells following the protocol of an ATP determination kit (Molecular Probes, Eugene, OR, USA) as described previously [30]. This assay was based on the luciferase requirement for ATP to produce 560 nm illuminant signals. A multilabel counter, obtained from BMG Labtech (Offenburg, Germany), was used to measure intensities of the illuminant light. Values were analyzed using the Gen5 software (vers. 3.03, BMG Labtech).

### 2.7. Quantification of Autophagic Cells

Proportions of autophagic cells were quantified by assessing acidic vesicular organelles in drug-resistant glioblastoma cells as described previously [23]. Following exposure to CoCl_2_, human and mouse drug-resistant glioblastoma cells at a density of 10^5^ cells/well were treated with 1 *μ*g/ml acridine orange for 20 min. After that, these TMZ-tolerant glioblastoma cells were harvested in DMEM without phenol red. A flow cytometer (Beckman Coulter, Fullerton, CA, USA) was used in this study to quantify levels of acridine orange with green and red fluorescence in glioblastoma cells. Intensities of fluorescent signals were analyzed using software from Beckman Coulter. 3-Methyladenine (3-MA), an inhibitor of autophagy, and rapamycin, an inducer of autophagy, were purchased from Sigma. 3-MA and rapamycin were dissolved in dimethyl sulfoxide (DMSO). After pretreatment with 1 mM 3-MA or 0.5 *μ*M rapamycin for 1 h, U87 MG-R and GL261-R glioblastoma cells were then exposed to CoCl_2_. Control cells received DMSO only.

### 2.8. Activities of Caspases-3 and -6

A fluorometric substrate assay was conducted to quantify activation of caspases-3 and -6 in human and mouse TMZ-resistant glioblastoma cells as described previously [31]. In brief, after CoCl_2_ treatment, human and mouse drug-resistant glioblastoma cells were lysed using a buffer containing Nonidet P-40 (1%), NaCl (200 mM), Tris/HCl (pH 7.4, 20 mM), leupeptin (10 mg/ml), aprotinin (0.27 U/ml), and phenylmethylsulfonyl fluoride (PMSF, 100 mM). Cell extracts were incubated with a specific fluorogenic peptide substrate at 50 mM in a cell-free system buffer containing HEPES (pH 7.4, 10 mM), mannitol (220 mM), sucrose (68 mM), NaCl (2 mM), KH_2_PO_4_ (2.5 mM), ethylene glycol tetraacetic acid (0.5 mM), MgCl_2_ (2 mM), pyruvate (5 mM), PMSF (0.1 mM), and dithiothreitol (1 mM). DEVD and VEID are specific peptide substrates for, respectively, detecting caspase-3 and -6 enzyme activities. For fluorescent detection, the DEVD and VEID substrates were conjugated with 7-amino-4-(trifluoromethyl)coumarin. A spectrometer (BMG Labtech) was used to measure intensities of the fluorescent products metabolized by caspases-3 and -6. Fluorescent values were examined using software from BMG Labtech and statistically analyzed.

### 2.9. Quantification of DNA Fragmentation

DNA fragmentation in human and mouse drug-sensitive and -resistant glioblastoma cells was quantified using a cellular ELISA kit (Boehringer Mannheim, Indianapolis, IN, USA) as described previously [32]. In brief, TMZ-tolerant glioblastoma cells were subcultured in 24-well tissue culture plates at a density of 2 × 10^5^ cells/well and labeled with BrdU for 12 h. Human and mouse glioblastoma cells were then harvested and suspended in culture medium. The cell suspension (100 *μ*l per well) was added to 96-well tissue culture plates. Drug-sensitive and -resistant glioblastoma cells were cultured under hypoxic conditions for various time periods in a humidified incubator with 5% CO_2_ at 37°C. Levels of BrdU-labeled DNA in the cytoplasm were measured with a microplate photometer (BMG Labtech) at 450 nm. The data of DNA fragmentation were then analyzed using software from BMG Labtech.

### 2.10. Assay of Apoptotic Cells

Proportions of drug-sensitive and -resistant glioblastoma cells under apoptotic insults were examined according to a previously described method [33]. After drug administration, glioblastoma cells were harvested and fixed in cold 80% ethanol. Following centrifugation and washing, fixed glioblastoma cells were stained with propidium iodide. A flow cytometer (Beckman Coulter) was used to measure fluorescent signals with a 560 nm dichroic mirror and a 600 nm bandpass filter. Intensities of these fluorescent signals in glioblastoma cells were quantified with software from Beckman Coulter.

### 2.11. Immunoblot Analysis

Immunoblot analyses were carried out to immunodetect levels of HIF-1*α*, LC3-I, LC3-II, PI3K, phosphorylated- (p-) and nonphosphorylated AKT and mTOR, vimentin, and *β*-actin in drug-resistant glioblastoma cells as described previously [34]. After exposure to hypoxia, lysates of drug-resistant glioblastoma cells were prepared in ice-cold radioimmunoprecipitation assay (RIPA) buffer, containing Tris-HCl (pH 7.2, 25 mM), Triton X-100 (1%), sodium dodecylsulfate (SDS, 0.1%), EDTA (1 mM), and NaCl (0.15 M). A mixture of proteinase inhibitors, viz., leupeptin (5 *μ*g/ml), sodium orthovanadate (1 mM), and PMSF (1 mM), was added to ice-cold RIPA buffer to prevent protein degradation. A bicinchonic acid protein assay kit purchased from Pierce (Rockford, IL, USA) was used to quantify protein concentrations. Cell lysates were loaded into SDS-polyacrylamide gel and electrophoretically separated. Then, the proteins were electrophoretically transferred to nitrocellulose membranes. Fiver percentage of on-fat milk was used to block the membranes at 37°C for 1 h. HIF-1*α* was immunodetected using a mouse monoclonal antibody (mAb) against human HIF-1*α* (Cell Signaling Technology, Danvers, MA, USA). LC3-I, LC3-II, PI3K, AKT, p-AKT, mTOR, and p-mTOR were recognized using mAbs or polyclonal antibodies (pAbs) purchased from Cell Signaling Technology. Cellular vimentin and *β*-actin proteins were immunodetected using mouse mAbs against human vimentin and mouse *β*-actin (Sigma), respectively. These immunoreactive protein bands were quantified with a digital imaging system (Syngene, Cambridge, UK) as described previously [35]. Intensities of these protein bands were analyzed using *β*-actin as the internal loading control.

### 2.12. HIF-1*α*-Knockdown

An RNA interference (RNAi) technique was applied in this study to knock down translation of HIF-1*α* as described previously [36]. HIF-1*α* small interfering (si) RNA (sc-35561), scrambled siRNA (sc-37007), and siRNA transfection medium (sc-36868) were purchased from Santa Cruz Biotechnology (Santa Cruz, CA, USA). At first, human TMZ-resistant glioblastoma cells were cultured in antibiotic-free DMEM and maintained in a humidified incubator with an atmosphere of 5% CO_2_ at 37°C for 24 h. After that, the HIF-1*α* siRNAs were diluted in siRNA transfection medium, and a HIF-1*α* siRNA duplex solution was added to the cells for transfection for 48 h. After replacing the old medium with normal DMEM, human U87 MG-R cells were exposed to CoCl_2_. Scrambled siRNA was applied as a negative control.

### 2.13. Statistical Analysis

Each value represses the mean ± standard deviation (SD) for at least three independent determinations. Statistical analyses were carried out using a two-way analysis of variance (ANOVA) and a post hoc Duncan's multiple-range test. Statistical differences were considered significant at *p* < 0.05.

## 3. Results

### 3.1. Selection and Preparation of Human and Mouse Drug-Resistant Glioblastoma Cells

**Figure 1:**
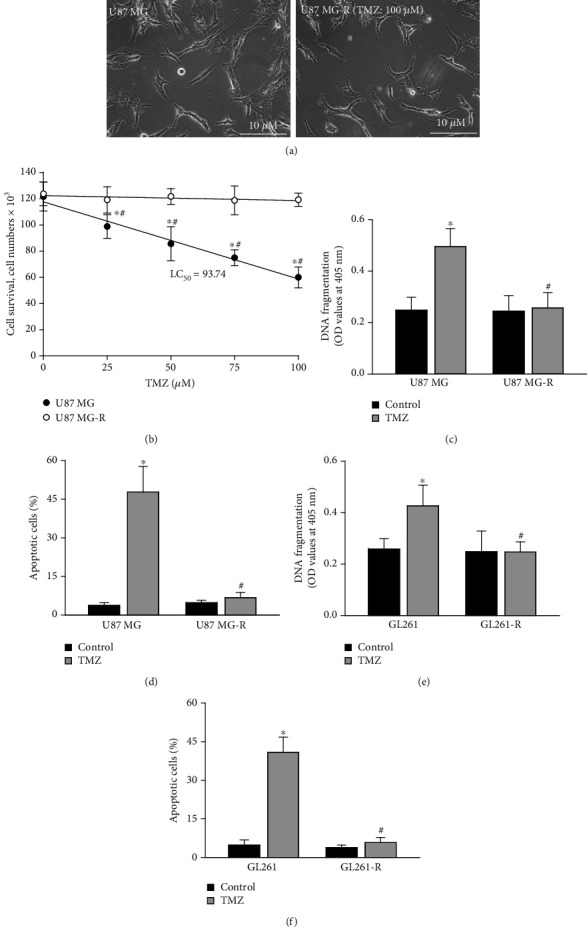
Selection and preparation of human drug-resistant glioblastoma cells. Human TMZ-tolerant U87 MG-R cells were selected from TMZ-sensitive U87 MG cells. (a) Morphologies of U87 MG and U87 MG-R cells are shown. Glioblastoma cells were exposed to TMZ at 25, 50, 75, and 100 *μ*M for 72 h. (b) Cell survival was assayed using a trypan blue exclusion method. (c, d) A cellular ELISA kit and a flow cytometric method were used to quantify DNA fragmentation and apoptotic cells, respectively. Murine GL261 and GL261-R glioblastoma cells were exposed to TMZ at 100 *μ*M. (e, f) DNA fragmentation and apoptotic cells were analyzed. Data are expressed as the mean ± SD for *n* = 6. ^∗^*p* < 0.05 vs. control and ^#^*p* < 0.05 vs. U87 MG.

Human and mouse glioblastoma cells that were resistant to TMZ treatment were prepared from their respective drug-sensitive brain tumor cells (Figure 1). No difference in morphologies of human drug-sensitive U87 MG and -resistant U87 MG-R cells was observed (Figure 1(a)). Exposure to TMZ at 25, 50, 75, and 100 *μ*M for 72 h caused significant 19%, 30%, 38%, and 51% decreases in survival of human U87 MG cells, respectively (Figure 1(b)). In contrast, treatment of human U87 MG-R glioblastoma cells with various concentrations of TMZ for 72 h did not change cell survival. Furthermore, exposure of U87 MG cells to 100 *μ*M TMZ for 72 h led to a significant 98% augmentation in DNA fragmentation (Figure 1(c)). The DNA integrity of U87 MG-R glioblastoma cells was not influenced by TMZ. Administration of TMZ at 100 *μ*M for 72 h led to a significant 48% elevation in apoptosis of human U87 MG cells (Figure 1(d)). At the same treated condition, TMZ did not trigger apoptosis of human U87 MG-R cells. Moreover, TMZ induced DNA fragmentation and cell apoptosis in drug-sensitive GL261 glioblastoma cells by 64% and 41%, respectively (Figures 1(e) and 1(f)). In comparison, treatment of mouse drug-resistant GL261-R glioblastoma cells with 100 *μ*M TMZ for 72 h did not trigger DNA fragmentation or cell apoptosis.

### 3.2. Exposure of Human and Mouse TMZ-Resistant Glioblastoma Cells to CoCl_2_ Increased Levels of HIF-1*α* and Led to Cell Death

**Figure 2:**
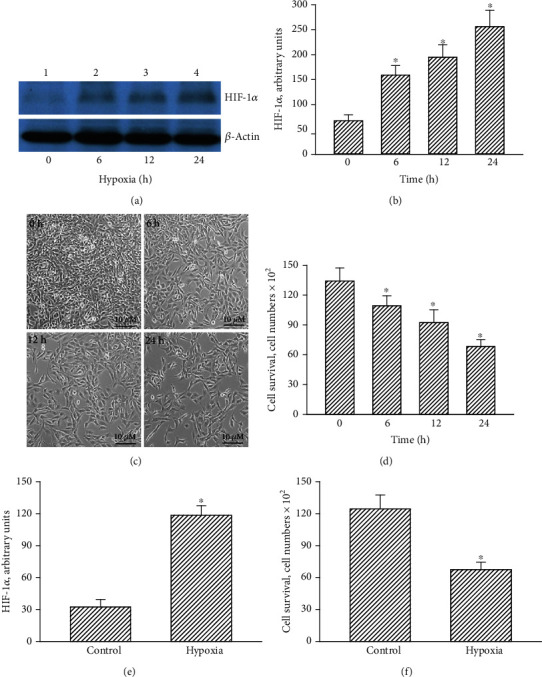
Effects of hypoxia induced by CoCl_2_ on levels of hypoxia-inducible factor- (HIF-) 1*α* and cell survival in human drug-resistant glioblastoma cells. Human drug-tolerant U87 MG-R cells were selected from TMZ-sensitive U87 MG cells. U87 MG-R cells were treated with hypoxia for 6, 12, and 24 h. (a) Levels of HIF-1*α* were immunodetected (top panel). *β*-Actin was analyzed as the internal control (bottom panel). (b) These protein bands were quantified and statistically analyzed. (c) Cell morphology was observed and photographed. (d) Cell survival was assayed with a trypan blue exclusion method. Mouse GL261-R glioblastoma cells were exposed to hypoxia for 24 h. (e, f) Levels of HIF-1*α* and cell survival were analyzed. Data are expressed as the mean ± SD for *n* = 6. ^∗^*p* < 0.05 vs. control.

Effects of CoCl_2_ on hypoxic insults to human TMZ-tolerant U87 MG-R cells were investigated (Figure 2). Treatment with 100 *μ*M CoCl_2_ for 6 h augmented levels of HIF-1*α* in U87 MG-R cells (Figure 2(a), top panel, lane 1). At 12 and 24 h after hypoxic treatment, HIF-1*α* in U87 MG-R cells were time-dependently elevated (lanes 3 and 4). *β*-Actin was measured as the internal loading standard (bottom panel). The protein band intensities were statistically analyzed (Figure 2(b)). Exposure to CoCl_2_ for 6, 12, and 24 h caused respective 2.4-, 2.9-, and 3.8-fold increases in levels of HIF-1*α* in human U87 MG-R glioblastoma cells. In comparison, exposure of human U87 MG-R cells to CoCl_2_ for 6, 12, and 24 h did not change levels of vimentin (Fig. S1). Compared to the untreated group, treatment with CoCl_2_ for 6 h reduced cell numbers (Figure 2(c)). After 12 and 24 h, hypoxia induced more death of human TMZ-tolerant glioblastoma cells. Moreover, our survival analysis showed that treatment of human U87 MG-R cells with 100 *μ*M CoCl_2_ for 6, 12, and 24 h, respectively, diminished cell survival by 19%, 31%, and 49% (Figure 2(d)). The HIF-1*α* levels in mouse GL261-R glioblastoma cells increased 3.6-fold following treatment with 100 *μ*M CoCl_2_ for 24 h (Figure 2(e)). Exposure of mouse GL261-R cells to CoCl_2_ led to a 46% reduction in cell survival (Figure 2(f)).

### 3.3. Hypoxia Induced by CoCl_2_ Triggered Mitochondrial Dysfunction, Proliferation Inhibition, and Cell Autophagy in Human and Mouse Drug-Resistant Glioblastoma Cells

**Figure 3:**
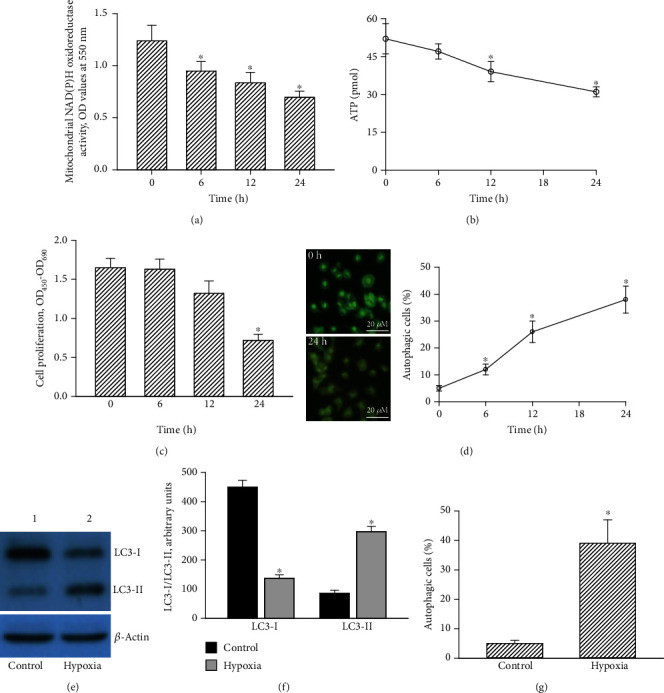
Effects of hypoxia induced by CoCl_2_ on mitochondrial NADH dehydrogenase activity, ATP levels, cell proliferation, cell autophagy, and levels of light chain (LC)3-I and LC3-2 in human drug-resistant glioblastoma cells. Human TMZ-tolerant U87 MG-R cells were selected from TMZ-sensitive U87 MG cells. U87 MG-R glioblastoma cells were treated with hypoxia for 6, 12, and 24 h. (a) Activity of mitochondrial NADH dehydrogenase was assayed using a colorimetric method. (b) Levels of ATP were measured using a bioluminescence assay. (c) Cell proliferation was measured by a thymidine incorporation assay. (d) Autophagic cells with acidic vesicular organelles were observed and photographed using a fluorescent microscope (left panel) and quantified with flow cytometry (right panel). (e) Levels of LC3-I and LC3-II were immunodetected (top panels). *β*-Actin was measured as the internal control (bottom panels). (f) These protein bands were quantified and statistically analyzed. Mouse GL261-R glioblastoma cells were exposed to hypoxia for 24 h. (g) Autophagic cells were quantified. Data are expressed as the mean ± SD for *n* = 6. ^∗^*p* < 0.05 vs. control.

Effects of the CoCl_2_-induced hypoxia insults to mitochondrial functions, cell proliferation, and cell autophagy were consecutively determined (Figure 3). Activities of mitochondrial NAD(P)H enzymes in U87 MG-R cells decreased by 23%, 32%, and 44% following respective exposure to CoCl_2_ for 6, 12, and 24 h (Figure 3(a)). Subsequently, treatment with CoCl_2_ for 12 and 24 h led to respective 25% and 40% reductions in cellular ATP levels (Figure 3(b)). Moreover, at 24 h after hypoxic administration, proliferation of human TMZ-resistant glioblastoma cells had dropped by 56% (Figure 3(c)).

Remarkably, exposure of human drug-tolerant glioblastoma cells to CoCl_2_ for 24 h induced the cells with acidic vesicles organelle (Figure 3(d), left panel). A flow cytometric examination revealed that exposure to CoCl_2_ for 6 h increased acidic vesicular organelles in human drug-tolerant glioblastoma cells by 12% (right panel). Following CoCl_2_ treatment for 12 and 24 h, autophagic cells were, respectively, increased 26% and 38%. An immunoblot image shows that LC3-I and -II in untreated U87 MG-R cells were detected (Figure 3(e), top panels, lane 1). Administration of CoCl_2_ for 24 h decreased levels of LC3-I but increased amounts of LC3-II (lane 2). *β*-Actin was immunodetected as the internal loading standard (bottom panel). Hypoxic treatment decreased of LC3-I by 69% but increased of LC3-II by 240% in TMZ-resistant glioblastoma cells (Figure 3(f)). Meanwhile, the CoCl_2_-induced hypoxia caused 39% of mouse GL261-R cells to undergo autophagy (Figure 3(g)).

### 3.4. CoCl_2_ Induced Hypoxic Insults to Human Drug-Resistant Glioblastoma Cells via a HIF-1*α*-Dependent Mechanism

**Figure 4:**
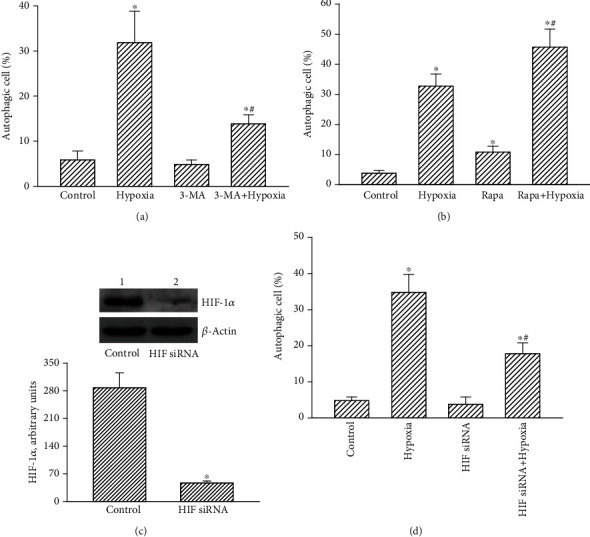
Effects of 3-methyladenine (3-MA), rapamycin (Rapa), and hypoxia-inducible factor- (HIF-) 1*α* knockdown on autophagy of human drug-resistant glioblastoma cells. Human temozolomide- (TMZ-) resistant U87 MG-R glioblastoma cells were selected from TMZ-sensitive U87 MG cells. (a, b) Human U87 MG-R glioblastoma cells were pretreated with 3-MA at 1 mM or Rapa at 0.5 *μ*M for 1 h and then exposed to hypoxia for additional 24 h. Control cells received DMSO only. A flow cytometric method was carried out to quantify autophagic cells. (c) Human U87 MG-R cells were treated with HIF-1*α* small interfering (si) RNA (HIF siRNA) for 48 h. Scrambled siRNA was applied to control cells as the negative control (control). HIF-1*α* was immunodetected, and *β*-actin was analyzed as the internal control. These protein bands were quantified and statistically analyzed. (d) Human U87 MG-R cells were pretreated with HIF-1*α* siRNA and then exposed to hypoxia. Autophagic cells were quantified using flow cytometry. Data are expressed as the mean ± SD for *n* = 6. ^∗^*p* < 0.05 vs. control and ^#^*p* < 0.05 vs. U87 MG.

Loss- and gain-of-function strategies were conducted to confirm the effects of the CoCl_2_-induced hypoxia on autophagic insults to TMZ-tolerant glioblastoma cells (Figures 4(a) and 4(b)). Administration of CoCl_2_ induced autophagy of human drug-resistant U87 MG-R cells by 32% (Figure 4(a)). In the control group, pretreatment with 3-MA alone did not trigger cell autophagy. However, administration of 3-MA attenuated hypoxia-induced autophagic insults to human U87 MG-R cells by 69% (Figure 4(a)). In contrast, pretreatment of human drug-tolerant glioblastoma cells with rapamycin did not influence cell autophagy (Figure 4(b)). Nevertheless, pretreatment with rapamycin increased hypoxia-induced autophagic insults to U87 MG-R cells by 45%.

At the same time, roles of HIF-1*α* in CoCl_2_-induced autophagy of human drug-resistant U87 MG-R glioblastoma cells were further investigated (Figures 4(c) and 4(d)). Application of HIF-1*α* small interfering (si) RNA to human U87 MG-R cells for 48 h caused obvious attenuation of HIF-1*α* levels compared to untreated cells (Figure 4(c), top panel). Protein bands were quantified using *β*-actin as the loading control, and the data were statistically analyzed. After application of HIF-1*α* siRNA, levels of HIF-1*α* in human U87 MG-R cells were reduced by 83% (Figure 4(c), bottom panel). The CoCl_2_-induced hypoxia triggered 35% of U87 MG-R cells undergoing autophagy (Figure 4(d)). Application of HIF-1*α* siRNA to human TMZ-resistant glioblastoma cells did not trigger cell autophagy. In comparison, knocking down HIF-1*α* translation concurrently suppressed 57% of hypoxia-induced autophagic insults to human U87 MG-R cells (Figure 4(d)).

### 3.5. The CoCl_2_-Induced Hypoxia Sequentially Decreased Levels of PI3K and Subsequent Phosphorylation of AKT and mTOR in Human Drug-Resistant Glioblastoma Cells

**Figure 5:**
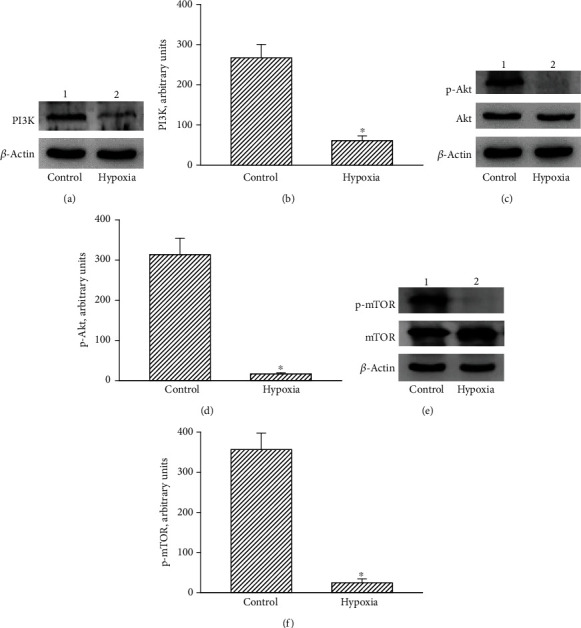
Signal-transducing mechanisms involved in hypoxia-induced autophagy of human drug-resistant glioblastoma cells. Human TMZ-tolerant U87 MG-R cells were selected from TMZ-sensitive U87 MG cells. Human U87 MG-R cells were treated with hypoxia for 24 h. (a, c, e) Levels of phosphoinositide 3-kinase (PI3K), phosphorylated- (p-) AKT, and p-mammalian target of rapamycin (mTOR) were immunodetected (top panels). *β*-Actin, AKT, and mTOR were analyzed as the internal controls for detection of PI3K, p-AKT, and p-mTOR, respectively (bottom panels). (b, d, f) These immunorelated protein bands were quantified and statistically analyzed. Data are expressed as the mean ± SD for *n* = 6. ^∗^*p* < 0.05 vs. control.

Molecular mechanisms of CoCl_2_-induced insults to human TMZ-tolerant glioblastoma cells were further investigated (Figure 5). In the control group, PI3K was immunodetected in human U87 MG-R glioblastoma cells (Figure 5(a), top panel, lane 1). In contrast, administration of CoCl_2_ to human glioblastoma cells obviously decreased levels of PI3K (lane 2). Intensities of these protein bands were measured using *β*-actin as a loading standard (bottom panel), and the data were statistically analyzed (Figure 5(b)). Exposure to hypoxia caused a 77% reduction in PI3K levels in human U87 MG-R cells (Figure 5(b)). Consecutively, AKT phosphorylation in U87 MG-R cells was alleviated following exposure to CoCl_2_ compared to the control group (Figure 5(c), top panel). AKT and *β*-actin were measured as internal controls (bottom two panels). The CoCl_2_-induced hypoxia diminished phosphorylation of AKT in human TMZ-resistant glioblastoma cells by 89% (Figure 5(d)). Consequently, hypoxia reduced mTOR phosphorylation in human U87 MG-R cells (Figure 5(e), top panel). mTOR and *β*-actin were measured as the internal controls (bottom two panels). Exposure of human drug-tolerant glioblastoma cells to hypoxia led to 91% repression of mTOR phosphorylation (Figure 5(f)).

### 3.6. Hypoxia Induced by CoCl_2_ Triggered Autophagy and Subsequent Apoptosis of Human and Mouse Drug-Resistant Glioblastoma Cells

**Figure 6:**
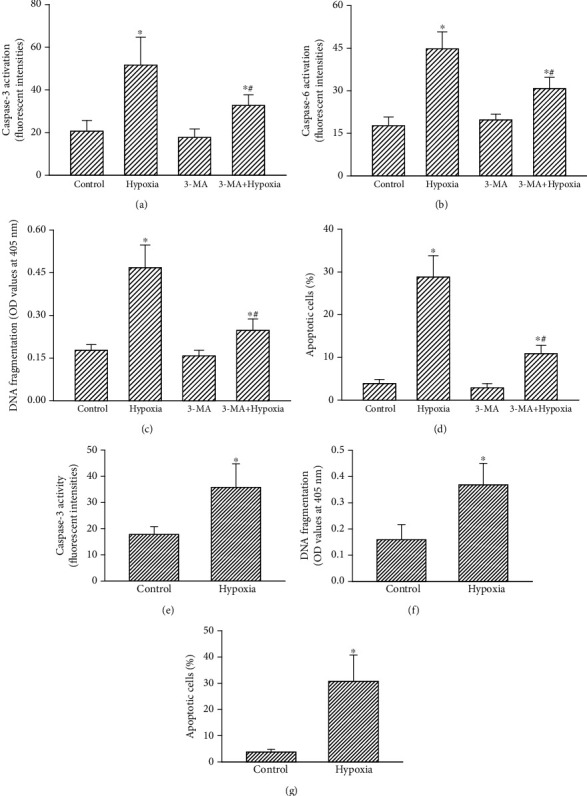
Effects of 3-MA on hypoxia-induced cascade activation of caspases-3 and -6, DNA fragmentation, and cell apoptosis in human drug-resistant glioblastoma cells. Human U87 MG-R glioblastoma cells were pretreated with 1 mM 3-MA for 1 h. Then, the cells were treated with hypoxia for 24 h. (a, b) Cascade activation of caspase-3 and caspase-6 were examined with a fluorometric substrate assay. (c, d) DNA fragmentation and apoptotic cells were analyzed. Mouse GL261-R glioblastoma cells were exposed to hypoxia for 24 h. (e–g) Caspase-3 activity, DNA fragmentation, and apoptotic cells were assayed. Data are expressed as the mean ± SD for *n* = 6. ^∗^*p* < 0.05 vs. control and ^#^*p* < 0.05 vs. U87 MG.

Treatment of human drug-resistant glioblastoma cells with CoCl_2_ enhanced the activity of caspase-3 by 2.5-fold (Figure 6(a)). Pretreatment with 3-MA did change activation of caspase-3. Nonetheless, 3-MA lowered hypoxia-induced caspase-3 activation by 62% (Figure 6(a)). Sequentially, caspase-6 activity in human U87 MG-R glioblastoma cells increased 2.5-fold (Figure 6(b)). Pretreatment with 3-MA alone did not affect caspase-6 activity but attenuated CoCl_2_-triggered activation of caspase-6 by 52%. Exposure to CoCl_2_ led to a 2.5-fold induction of DNA fragmentation in U87 MG-R cells (Figure 6(c)). In parallel, hypoxia triggered 29% of human U87 MG-R cells to undergo apoptosis (Figure 6(d)). Pretreatment with 3-MA did not affect the DNA integrity or cell apoptosis. In contrast, pretreatment of human TMZ-tolerant glioblastoma cells with 3-MA caused significant 76% and 72% depressions in hypoxia-induced DNA fragmentation and cell apoptosis, respectively (Figures 6(c) and 6(d)). In addition, administration of hypoxia augmented caspase-3 activities by twofold in mouse GL261-R cells (Figure 6(e)). Subsequently, exposure of mouse drug-resistant GL261-R cells to CoCl_2_ caused a significant 2.3-fold stimulation of DNA fragmentation (Figure 6(f)). Accordingly, apoptotic insults to mouse drug-resistant glioblastoma cells were induced by 31% after CoCl_2_ administration (Figure 6(g)).

### 3.7. Exposure to Hypoxia for 96 h Induced Apoptotic Insults to Human Drug-Resistant Glioblastoma Cells without Affecting Human Normal Astrocytes

**Figure 7:**
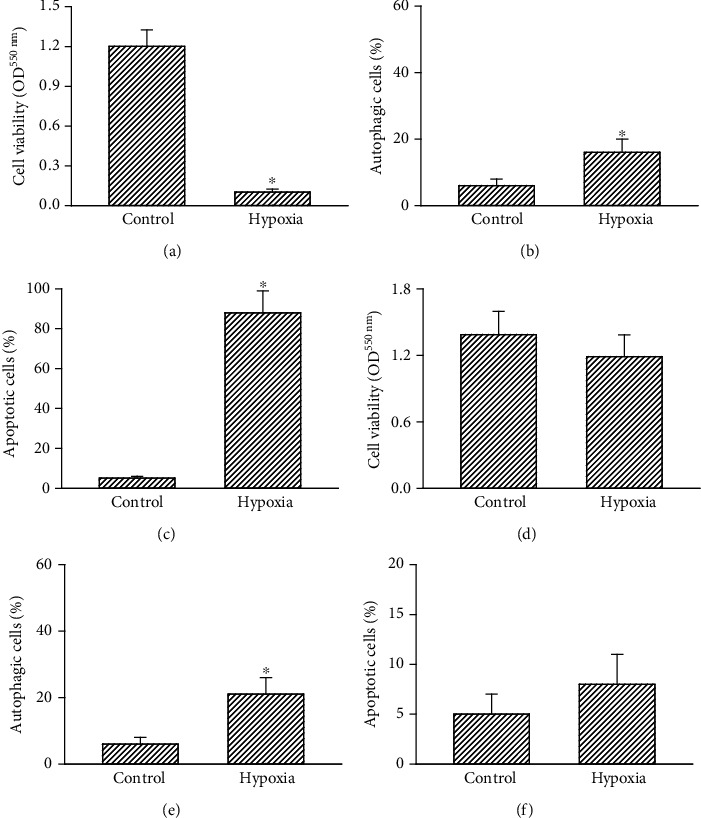
Effects of CoCl_2_ treatment for 96 h on viability, autophagy, and apoptosis of human drug-resistant glioblastoma cells and normal astrocytes. (a–c) Human temozolomide- (TMZ-) resistant U87 MG-R glioblastoma cells and (d–f) human HA-h astrocytes were exposed to CoCl_2_ for 96 h. (a, d) Cell viability was assayed using a colorimetric method. (b, e) Autophagic and (c, f) apoptotic cells were quantified using flow cytometry. Each value represents the mean ± SD for *n* = 3. The symbol ^∗^ indicates that a value significantly (*p* < 0.05) differed from the respective control group.

Treatment of human U87 MG-R cells to CoCl_2_ for 96 h decreased cell viability by 92% (Figure 7(a)). In addition, exposure to CoCl_2_ for 96 h caused 16% and 88% of human drug-resistant glioblastoma cells undergoing autophagy and apoptosis, respectively (Figures 7(b) and 7(c)). The safety of CoCl_2_ to human normal HA-h astrocytes was then evaluated (Figures 7(d)–7(f)). Exposure of HA-h cells to 100 *μ*M CoCl_2_ for 96 h did not influence cell viability (Figure 7(d)). In contrast, treatment with CoCl_2_ for 96 h led to a slight 21% induction of HA-h cells undergoing autophagy but did not trigger apoptotic insults (Figures 7(e) and 7(f)).

## 4. Discussion

Administration of CoCl_2_ can induce hypoxic conditions and consequent autophagic apoptosis of drug-resistant glioblastoma cells. In this study, we demonstrated that exposure to CoCl_2_ could trigger hypoxic stress to human and murine TMZ-resistant glioblastoma cells. In parallel, hypoxic conditions disrupted mitochondrial ATP synthesis and induced death of cells of these two drug-resistant glioblastoma cell lines. In addition, hypoxia suppressed proliferation of human TMZ-tolerant glioblastoma cells. GBM is the commonest and most aggressive brain tumor [1]. Inopportunely, GBM patients have very poor prognoses because most patients eventually have become drug-resistant and recurrent [3]. To the present, TMZ is routinely used as the first-line drug for treatment of GBM patients [2]. The malignance of glioblastomas can be elucidated because following surgery, residual glioblastoma cells can rapidly proliferate, migrate, and invade to the other sites for development of new brain tumors. Hypoxia is able to suppress proliferation and viability of drug-sensitive glioblastoma cells [25]. In the present study, we further identified the beneficial actions of the CoCl_2_-induced hypoxic conditions to suppress proliferation and survival of TMZ-resistant glioblastoma cells. As to the mechanisms, administration of hypoxic stress meaningfully induced autophagy and subsequent apoptosis of human and murine drug-resistant glioblastoma cells. In response to malnutrition, cells can temporarily survive by activating a process of self-degradation and catabolism, called autophagy [17]. Autophagic cells will subsequently either survive or proceed to necrosis or apoptosis [17, 18]. Furthermore, autophagy was also shown to be involved in the prevention of certain diseases, including tumors [26]. The drug-resistant glioblastoma cells highly defend against apoptosis. Recently, we demonstrated advantages of a longer period of hypoxia induced by honokiol, a multifunctional antitumor drug, on the killing of human neuroblastoma cells and glioblastoma cells via an autophagic apoptosis pathway [23–25]. Recently, autophagic cell death has attracted researchers as a potential method for cancer therapy. In this study, we provide serial evidence to show the benefits of CoCl_2_-induced hypoxia of killing drug-resistant glioblastoma cells through activating an autophagic and subsequent apoptotic mechanism. As a result, longer hypoxia induced by certain agents such as CoCl_2_ or honokiol may be clinically applied as a *de novo* strategy for treating chemoresistance in malignant and recurrent glioblastomas via an autophagic apoptosis pathway.

Administration of CoCl_2_ led to hypoxic stress and consequently induced insults to human and murine TMZ-resistant glioblastoma cells. Drug-resistant glioblastoma cells used in this study were prepared according to a continuous selection protocol described in our previous study [37]. Compared to chemosensitive human U87 MZ and mouse GL261 cells, these two TMZ-resistant U87 MZ-R and GL261-R cell lines have similar morphologies. Nevertheless, administration of TMZ induced apoptotic insults to human and mouse TMZ-sensitive glioblastoma cells but did not affect chemoresistant cells. Fascinatingly, exposure to CoCl_2_ time-dependently raises levels of HIF-1*α* in drug-resistant glioblastoma cells. In the hypoxic microenvironment, HIF-1/2*α*, two transcriptional factors, can be massively induced to regulate certain gene expressions in response to oxygen deficiency-induced stress [16]. CoCl_2_ can chelate Fe^2+^ ions in hemoglobin to decrease the oxygen supply to cells [38]. Additionally, administration of CoCl_2_ raises levels of cellular HIF-1*α* by inhibiting the activity of prolyl-4-hydroxylase, a HIF-1*α*-specific proteinase [39]. Thus, CoCl_2_ can elevate levels of HIF-1*α* in human and mouse TMZ-resistant glioblastoma cells and induce intracellular hypoxic stress. At the same time, the CoCl_2_-induced hypoxia diminished proliferation and survival of drug-resistant glioblastoma cells. In tumorigenesis, hypoxia can stimulate the proliferation of tumor cells via a HIF-1*α*-dependent transcriptional mechanism [40]. Nonetheless, Dai et al. reported that in a CoCl_2_-induced hypoxic microenvironment, proliferation and viability of PC-2 cells were lessened, and the cells underwent apoptosis [41]. Our previous studies also demonstrated the oppressive effects CoCl_2_ on the proliferation and survival of drug-sensitive glioblastoma cells [25]. In parallel, enzyme activity of mitochondrial NAD(P)H oxidoreductase and levels of cellular ATP in human TMZ-resistant glioblastoma cells were repressed following exposure to hypoxia. Thus, the CoCl_2_-induced hypoxia suppressed proliferation and survival of drug-resistant glioblastoma cells via lowering mitochondrial ATP synthesis. However, the reasons explain the relation between ATP reductions on suppression of cell proliferation in hypoxia-treated drug-resistant glioblastoma cells need to be further investigated.

Hypoxia induced by CoCl_2_ can trigger autophagy of human and murine drug-resistant glioblastoma cells via a HIF-1*α*-dependent pathway. Our flow cytometric analysis of acidic vesicular organelles in human and mouse drug-tolerant glioblastoma cells revealed that administration of CoCl_2_ time-dependently induced autophagic insults. Simultaneously, the ratio of LC3-II over LC3-I significantly increased after exposure to CoCl_2_. When the cells are undergoing autophagy, acidic vesicular organelles were formed [23, 42]. At the same time, the ratio of LC3-II over LC3-I was enhanced. In this study, we further used gain- and loss-of-function strategies to confirm the hypoxia-induced autophagy of TMZ-resistant glioblastoma cells. As usual, 3-MA and rapamycin were applied as a respective inhibitor and an inducer of cell autophagy [43]. After administration of 3-MA and rapamycin to chemoresistant glioblastoma cells, hypoxia-induced autophagic insults were, respectively, attenuated and enhanced. Thus, multiple lines of evidence showed the action of the CoCl_2_-induced hypoxia in inducing autophagy of drug-resistant glioblastoma cells, thereby inducing autophagic insults to TMZ-resistant glioblastoma cells. More interestingly, knocking down HIF-1*α* concurrently lowered CoCl_2_-induced autophagic insults to human TMZ-resistant glioblastoma cells. HIF-1*α* can induce cell autophagy via inducing BNIP3 and LC3 expressions [22]. In addition, a previous study reported that prolonged hypoxia induced mitochondrial autophagy via activation of a HIF-1*α*/BINP3/Beclin-1/Atg5 mechanism [44]. In the present study, exposure to CoCl_2_ led to consequent mitochondrial dysfunction. As a result, one possible mechanism explaining CoCl_2_-induced autophagy of human drug-resistant glioblastoma cells is via triggering HIF-1*α*-dependent mitochondrial autophagy. Being a potential target for cancer therapy, autophagy has recently attracted attention of oncologic physicians and researchers [45]. Chemoresistance and recurrence are two critical factors driving malignance and poor prognoses of GBM patients [6]. In this study, we provide *in vitro* evidence to demonstrate the potential effects of prolonged hypoxia induced by CoCl_2_ for treating GBM by inducing autophagic insults to drug-resistant glioblastomas.

Hypoxia induced by CoCl_2_ led to autophagy of human drug-resistant glioblastoma cells through targeting the PI3K-AKT-mTOR pathway. After exposure to CoCl_2_, levels of PI3K in human TMZ-resistant glioblastoma cells were significantly diminished. In tumorigenesis, PI3K is genetically overexpressed or mutated in the brain, breasts, prostate, stomach, colon, and endometrium [46]. So, targeting PI3K was investigated as a new strategy for treating various types of tumors such as breast cancer [47]. AKT is a downstream target of PI3K. Our present data reveal that treatment with CoCl_2_ decreased levels of AKT in human drug-tolerant glioblastoma cells. Hence, the hypoxia-induced downregulation of AKT was due to suppression of PI3K production. Inhibition of the PI3K/AKT pathway is recognized as a new weapon for fighting cancer incidence [46]. Our present data prove the suppressive effects of CoCl_2_ against the proliferation of human TMZ-resistant glioblastoma cells. Thus, the hypoxia-induced blockage of the PI3K-AKT pathway may be beneficial for inhibiting the growth of chemoresistant glioblastomas. mTOR, a serine/threonine protein kinase, plays a crucial role in the balance between catabolism and anabolism [48]. Phosphorylation of mTOR, activated by the PI3K-AKT pathway, can drive cellular catabolism and depress cell autophagy [49]. In parallel with an interruption of the PI3K/AKT pathway, CoCl_2_ weakened phosphorylation of mTOR in human TMZ-resistant glioblastoma cells. Hence, one possible mechanism explaining the CoCl_2_-induced reduction in levels of phosphorylated mTOR in TMZ-resistant glioblastomas is due to disruption of the PI3K-AKT pathway. In addition to HIF-1*α*, HIF-2*α* is another factor that can be upregulated by hypoxia [16]. Under hypoxic conditions, the proteasome-dependent stability of HIF-1/2*α* is involved in regulation of tumor-induced angiogenesis and metastasis via the PI3K/AKT pathway [50]. In aggressive neuroblastomas, Mohlin et al. reported that suppression of HIF-2*α* by targeting PI3K/mTORC1 can improve therapeutic efficacy [51]. This study demonstrated that knocking down HIF-1*α* simultaneously attenuated hypoxia-induced cell autophagy. Therefore, the CoCl_2_-induced hypoxia can trigger autophagic insults to drug-resistant glioblastoma cells via targeting the PI3K-AKT-mTOR pathway.

Hypoxia induced by CoCl_2_ triggered autophagic apoptosis of human and murine drug-resistant glioblastoma cells. Prolonged exposure to CoCl_2_ of human and murine TMZ-resistant glioblastoma cells induced cascade activation of caspases-3 and -6, DNA fragmentation, and cell cycle arrest at the sub-G_1_ phase. Caspase activation, DNA fragmented damage, and cell cycle arrest are characteristic features indicating that cells are undergoing apoptosis [52, 53]. Interestingly, pretreatment of human TMZ-resistant glioblastoma cells with 3-MA reduced hypoxia-induced autophagy. At the same time, CoCl_2_-induced cascade activation of caspases-3 and -6, DNA breakage, and apoptosis in human TMZ-tolerant glioblastoma cells were significantly lowered following pretreatment with 3-MA. Autophagic cells will survive or proceed to die [17, 18]. Our present data showed that prolonged administration of CoCl_2_ can induce autophagic insults to TMZ-resistant glioblastoma cells, resulting in cell death via an apoptotic mechanism. Autophagic cell death is recognized as a separate form of cell death from cell apoptosis and necrosis [54]. Nonetheless, our present study showed that CoCl_2_ can trigger autophagic apoptosis of human TMZ-tolerant glioblastoma cells. Specific induction of apoptosis of tumor cells can be applied as an anticancer mechanism for cancer therapy [53]. However, GBM is a very aggressive tumor because it is usually hard to induce apoptosis in glioblastoma cells by chemotherapeutic drugs [55]. Therefore, hypoxia-induced autophagic apoptosis has the potential to serve as an alternative strategy for therapy of brain tumors.

## 5. Conclusions

**Figure 8:**
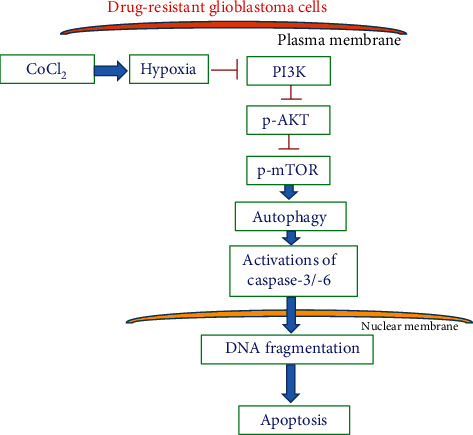
Proposed signal-transducing pathways of hypoxia-induced autophagy and subsequently apoptotic killing of human drug-resistant glioblastoma cells. Treatment of human TMZ-resistant glioblastoma cells with CoCl_2_ induces hypoxic insults by suppressing phosphoinositide 3-kinase- (PI3K-) involved signal-transducing phosphorylations of AKT and mammalian target of rapamycin (mTOR). Consequently, long-period administration of CoCl_2_ induced cascade activation of caspases-3 and -6, DNA breakage, and apoptotic insults to human drug-resistant glioblastoma cells.

In this study, we successfully selected human and mouse drug-resistant glioblastoma cells as our experimental models. Exposure of human and mouse TMZ-resistant glioblastoma cells to CoCl_2_ increased HIF-1*α* levels and induced hypoxic stress and insults (Figure 8). Subsequently, prolonged hypoxia induced by CoCl_2_ led to mitochondrial dysfunction. Interestingly, administration of hypoxia elevated proportions of drug-resistant glioblastoma cells with acidic organelles and the ratio of cellular LC3-II over LC3-I. Loss- and gain-of-function strategies were used to further demonstrate that pretreatment with 3-MA and rapamycin, respectively, attenuated and enhanced consequent CoCl_2_-induced cell autophagy. Importantly, knocking down HIF-1*α* translation using RNAi concurrently diminished CoCl_2_-induced cell autophagy. Thus, these manifold lines of evidence showed that prolonged hypoxia induced by CoCl_2_ could trigger hypoxic insults to human and mouse TMZ-resistant glioblastoma cells via a HIF-1*α*-dependent mechanism. As to the mechanisms, administration of CoCl_2_ decreased signal-transducing activation of PI3K and AKT (Figure 8). Successively, levels of phosphorylated mTOR in human drug-resistant glioblastoma cells were reduced by CoCl_2_. Fascinatingly, prolonged administration of hypoxia sequentially induced cascade activation of caspases-3 and -6, DNA fragmentation, and apoptotic insults in TMZ-tolerant glioblastoma cells (Figure 8). Using 3-MA to suppress CoCl_2_-induced autophagy simultaneously defended against apoptotic damage. Therefore, this study showed that prolonged hypoxia induced by CoCl_2_ can induce autophagic apoptosis of drug-resistant glioblastoma cells via suppression of the PI3K-AKT-mTOR pathway (Figure 8). To the present, chemoresistance and recurrence are the most serious issues and challenges for therapy of GBM patients. CoCl_2_-induced hypoxia and subsequent autophagic apoptosis may be a *de novo* strategy for treating glioblastomas. We are carrying out a translational study to further confirm our *in vitro* findings.

## Data Availability

All data generated or analyzed during this study are included in this article.

## Supplementary Materials

Supplementary materials Figure S1: effects of CoCl_2_ on levels of vimentin in human drug-resistant glioblastoma cells. Human TMZ-tolerant U87 MG-R cells were selected from TMZ-sensitive U87 MG cells. U87 MG-R glioblastoma cells were treated with hypoxia for 6, 12, and 24 h. (a) Levels of vimentin were immunodetected. *β*-Actin was analyzed as the internal control. (b) These immunorelated protein bands were quantified and statistically analyzed. Data are expressed as the mean ± SD for *n* = 3.
