# Supplementary figures and images for: Hypoxia Induced by Cobalt Chloride Triggers Autophagic Apoptosis of Human and Mouse Drug-Resistant Glioblastoma Cells through Targeting the PI3K-AKT-mTOR Signaling Pathway
Source: Oxid Med Cell Longev. 2021 May 27;2021:5558618. doi: 10.1155/2021/5558618 (PMC8177987; doi:10.1155/2021/5558618)

(a)

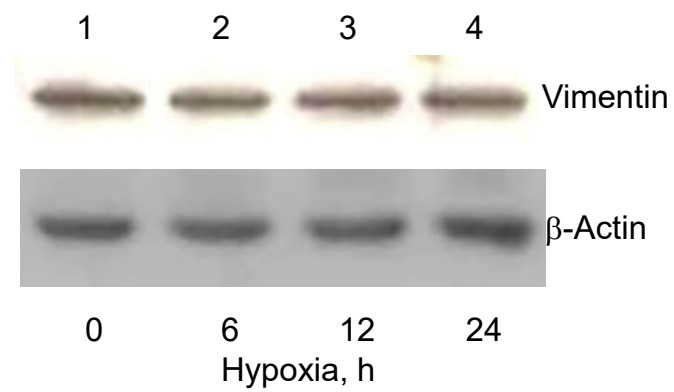

(b)

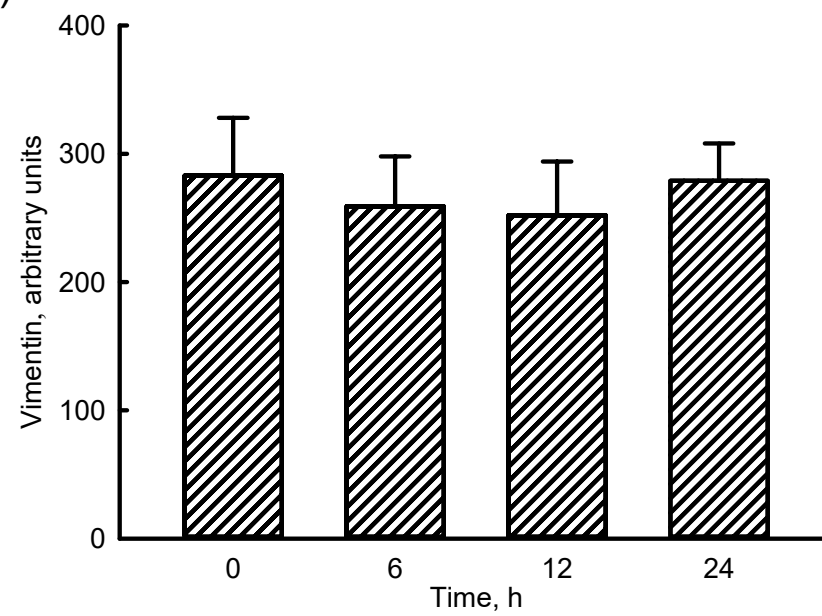

Supplement: Supplementary materials — Figure S1: effects of CoCl2 on levels of vimentin in human drug-resistant glioblastoma cells. Human TMZ-tolerant U87 MG-R cells were selected from TMZ-sensitive U87 MG cells. U87 MG-R glioblastoma cells were treated with hypoxia for 6, 12, and 24 h. (a) Levels of vimentin were immunodetected. β-Actin was analyzed as the internal control. (b) These immunorelated protein bands were quantified and statistically analyzed. Data are expressed as the mean ± SD for n = 3. [file 5558618.f1.pdf]
